# The Prevalence, Predictors, and Health Consequences of Disagreement in Reports of Child Maltreatment Exposure

**DOI:** 10.1007/s10578-024-01721-2

**Published:** 2024-05-30

**Authors:** Erin C. Dunn, Samantha C. Ernst, Kristen Nishimi, Kristen R. Choi

**Affiliations:** 1 Psychiatric and Neurodevelopmental Genetics Unit, Center for Genomic Medicine, Massachusetts General Hospital, Boston, MA USA; 2 Center On the Developing Child at Harvard University, Cambridge, MA USA; 3 Department of Psychiatry, Harvard Medical School, Boston, MA USA; 4 Mental Health Counseling and Behavioral Medicine Program, Boston University School of Medicine, Boston, MA USA; 5 Mental Health Service, San Francisco Veterans Affairs Health Care System, San Francisco, CA USA; 6 Department of Psychiatry and Weill Institute for Neurosciences, University of California San Francisco, San Francisco, CA USA; 7 School of Nursing, University of California Los Angeles, 700 Tiverton Ave, Los Angeles, CA 90095 USA; 8 Department of Health Policy and Management, Fielding School of Public Health, University of California Los Angeles, Los Angeles, CA USA

**Keywords:** Child maltreatment, Abuse, Trauma, Agreement, Depression

## Abstract

The purpose of this study was to examine the prevalence, predictors, and consequences of disagreement between prospective caregiver and retrospective child reports of childhood physical and emotional maltreatment. The design was a secondary analysis of data from the Avon Longitudinal Study of Parents and Children, a three-decade long UK-based birth cohort. Prospective caregiver reports were in poor to fair agreement with retrospective child reports for physical and emotional maltreatment exposure, with caregivers tending to underreport exposure. Disagreement between reporters was associated with increased risk of depressive symptoms and substance use severity, but decreased risk for mental health diagnoses. Screening measures of childhood maltreatment exposure should take caution against using measures from different reporters interchangeably (i.e., from mother versus child). Disagreement in reports may indicate unmet need for mental health evaluation.

**Supplementary Information:**

The online version contains supplementary material available at 10.1007/s10578-024-01721-2.

## Introduction

Childhood maltreatment is a strong predictor of poor health outcomes during childhood and across the lifespan, increasing disease risk by 2- to 6-fold [1–5]. Given the harm of child maltreatment, many clinicians and researchers are assessing child maltreatment exposure in clinical practice and research, as a means to identify high-risk populations, identify immediate safety issues, reduce long-term health risk, and understand the predictors and consequences of these experiences.

However, a lack of reliable tools to measure the occurrence of maltreatment has remained a major barrier to these efforts. Most often, researchers use self-reports, relying on *retrospective* study designs, wherein adolescents or adults report experiences from their childhood, or *prospective* study designs, in which a caregiver reports the child’s experiences over time. Most studies using self-reports of childhood maltreatment exposure experiences are retrospective in nature. Although there are also child-report measures available, caregiver reporting is often used in practice due to the developmental challenges of measurement with young children. These reporting types have limitations. Retrospective measures are susceptible to memory and recall biases, including infantile amnesia (when adults cannot recollect childhood episodic memories [6–8]. Furthermore, fear of legal consequence, denial, or shame, may skew prospective measures by parents toward underreporting, especially when the reporter has perpetrated the maltreatment or has a relationship with the perpetrator [9, 10].

Studies gathering reports of child maltreatment from multiple sources often find disagreement between informants [9, 11–14]. A recent meta-analysis of 16 different studies found poor agreement between prospective reports from various informants and retrospective self-reports, such that only about half of those identified retrospectively as having experienced maltreatment also had a concordant prospective report [15]. Similar studies have also observed that adverse health outcomes are more closely associated with retrospective versus prospective maltreatment reports [16–18].

Little is known about factors that drive child maltreatment reporting disagreement and how might reporter disagreement, in turn, shapes child health. Although there are known demographic and family factors associated with differences in maltreatment exposure (e.g., child sex, race, maternal mental health status, parent history of abuse or neglect, bonding), these factors have not been extensively studied in relation to reporting agreement. Without these insights, clinicians and researchers are unable to understand the benefits and drawbacks of different measurement approaches and identify the mechanisms through which different reporting types may contribute to adverse health outcomes. The knowledge gap in our understanding of the predictors and consequences of child maltreatment reporting disagreement also creates clinical practice challenges for deciding who should report maltreatment and what sources should be considered for reliable measurement and appropriate response.

To address these gaps, we investigated the prevalence, predictors, and consequences of disagreement between prospective and retrospective reports of child maltreatment using data from the Avon Longitudinal Study of Parents and Children (ALSPAC). First, we examined levels of agreement on reports of physical maltreatment (PM) and emotional maltreatment (EM) exposure by comparing: (1) prospective reports from mothers compared to prospective reports from her partner; and (2) prospective caregiver reports (combining mother and partner reports) compared to retrospective child reports. We hypothesized that (1) mothers would report maltreatment more frequently than their partners; and (2) children would report maltreatment more frequently than their caregivers. Second, we explored the predictors of disagreement between these two pairs of reporters. This aim was exploratory and thus no hypotheses were specified. Third, we examined the extent to which disagreement between reporters was associated with child health outcomes in young adulthood. We hypothesized that disagreement would be associated with greater health risk. The ALSPAC sample is uniquely poised to examine these questions, given its 30-year duration of follow-up, often with repeated measures from multiple family members [19].

## Methods

### Sample

ALSPAC is a birth cohort from Avon, England that follows pregnant mothers whose children had expected delivery dates between April 1991 and December 1992 [19, 20]. Informed consent for the use of data collected was obtained from participants following the recommendations of the ALSPAC Ethics and Law Committee at the time (additional details about the sample and methods are available in Supplemental Materials).

We constructed two analytic samples from the data (Fig. S1). Our primary analytic sample included reporter pairs from both mother and partner with three or more completed timepoints (Table S1) in which they responded to questions regarding maltreatment behaviors (N = 5799 pairs) [21]. Our secondary analytic sample was restricted to pairs from the primary sample who also had child-reported data for maltreatment questions as reported at age 22; this secondary sample allowed us to examine the differences in reports of maltreatment by caregivers (combined mother and partner reports) and children (N = 2373 pairs) [21]. See Table S2 for comparisons between our analytic subsamples and the total sample.

### Measures

We examined two types of maltreatment behaviors using reports from mail-in questionnaires sent to mothers, partners, and children. At seven different timepoints starting at child aged 8 months and ending at 9 years, mothers and partners reported on maltreatment exposure using an ALSPAC-designed measure (Table S3 for details). Most participants (≥ 92%) completing the partner report identified as the child’s father at each reporting time for both analytic samples (Table S4). We derived two variables to classify children as exposed or unexposed to PM and EM, respectively. Exposure to maltreatment was defined as at least one affirmative response by the reporter to one item in each maltreatment category, regardless of the perpetrator(s) identified. Exposure to “caregiver” maltreatment behavior was defined as maltreatment reported by *either* the mother or partner, regardless of the perpetrator(s) identified.

At age 22, children reported their exposure to PM and EM by an adult in the family before age 11 (see Table S3 for questionnaire details). The questions were derived from the psychometrically-validated Child Abuse Questionnaire and Sexual Experiences Survey [22, 23]. Each item was rated on a 5-point frequency scale: never; rare; sometimes; often; or very often. For our analyses, children were considered exposed to maltreatment if their response was greater than “never” for any of the items associated with PM and EM (sensitivity analyses with other cut-points are described later).

### Predictors of Disagreement

Disagreement was defined as when the mother reported exposure while her partner did not (or vice versa) and when the caregivers reported exposure while child did not (or vice versa). We examined 16 possible predictors of reporter disagreement. The following demographic characteristics were investigated, because they are risk factors of child maltreatment exposure [24, 25]: child’s sex, child’s race, and maternal factors (education level, marital status, age at child’s birth, and number of previous pregnancies). We also investigated familial factors as predictors of disagreement given their association with child maltreatment exposure, including maternal postnatal depression, caregivers’ exposure to neglect and abuse as children, maternal mental health history, and maternal and paternal bonding with child (see Sect. "Results" of Supplemental Materials for variable coding and complete list of variables) [26–28].

### Child Health Outcomes

Maltreatment has been associated with a wide range of health outcomes. Thus, we broadly evaluated the consequences of discordant reports, using data on both objective and subjective health measures of the children collected at two timepoints: (a) a self-report mail-in questionnaire at 22 years, and (b) a clinic-based assessment at age 24 years. Health outcomes included self-reported lifetime presence of 8 conditions (Sect. "Discussion" of Supplemental Materials), general health quality, as well as clinically assessed body mass index and blood pressure. Mood/behavioral outcomes included self-reported lifetime presence of four conditions (Sect. "Discussion" of Supplemental Materials), depressive symptoms at age 22, and clinically assessed symptoms of depression, anxiety, and substance abuse at the 24-year timepoint. By using data from both timepoints we could better ascertain the implications related to reporter discordance and maintain temporality in our exposure–outcome association.

### Primary Analyses

First, to examine the level of agreement across reports of child maltreatment, we calculated two agreement statistics: the kappa coefficient (κ) of agreement and the prevalence-adjusted bias-adjusted κ (PABAK). κ is often used to assess the level of inter-rater reliability and is useful in situations where there is no standard measure to estimate validity [29]. However, some argue against using the κ coefficient, particularly when an outcome is rare, as rare events can create the paradox of high concordance but low κ values [30, 31]. Because we expected maltreatment to be underreported and thus rare in this population-based sample, which could artificially decrease κ, we also calculated the PABAK.^31^ PABAK, like κ, provides an estimate of inter-rater agreement, but it also adjusts for the prevalence of the outcome. κ and PABAK values range from 0 (no agreement) to 1 (perfect agreement) and were interpreted as degrees of agreement, per standard interpretation guidelines: (a) 0.01–0.20 (slight); (b) 0.21–0.40 (fair); (c) 0.41–0.60 (moderate); and (d) > 0.60 (substantial) [32].

Second, we examined demographic and familial predictors of disagreement between reports of child maltreatment. Disagreement was coded as a binary variable capturing the pair discordance versus concordance for maltreatment presence (0 = reporter pair agreed on presence/absence of maltreatment; 1 = reporter pair disagreed). We used simple logistic regression to model the association between demographic and familial factors as predictors of disagreement between: (a) mother and partner reports and (b) caregiver and child reports. No covariates were included in these analyses, as this was the first study to test these associations. Finally, we examined whether disagreement between caregiver and child reports was associated with child health outcomes using simple logistic and linear regression. Because we examined bivariate associations between disagreement and multiple, potentially correlated child outcomes, we report both unadjusted and Bonferroni adjusted p-values corrected for multiple testing. All analyses were conducted using SAS®.

Given the results of prior studies suggesting there are distinct pathways between prospective and retrospective reporting with health outcomes, we also explored, for comparison, associations between prospective caregiver-reported and retrospective child-reported maltreatment exposure and the health outcomes described above.

### Sensitivity Analysis

To determine how results might change if different cut-points were applied to define child-reported maltreatment from the frequency rating scales, we re-analyzed the primary analyses using a more conservative definition of child-reported exposure (maltreatment items occurring “often” or “very often”) (Sect. "Methods" of Supplemental Materials).

## Results

### Prevalence of Disagreement

The most common response combination from mother–partner pairs was for agreement that their child had not been exposed to PM (93.5%, N = 5420) or EM (84.3%, N = 4891, Table 1). Pair-wise disagreement was rarer: 3.1% of pairs (N = 182) had PM reported by mothers, but not partners; 2.6% of partners (N = 151) reported PM, but not mothers; 7.2% of pairs (N = 418) had EM reported by mothers, but not partners; and 5.7% of partners (N = 328) reported EM, but not mothers. While κ coefficients showed slight agreement between mothers and partners for PM (κ = 0.19) and EM (κ = 0.23), the PABAK values indicated substantial agreement by pairs on reports of PM (PABAK = 0.89) and EM (PABAK = 0.74, Table 1).

**Table 1:** Prevalence and agreement between prospective mother and partner reports of their child’s exposure to physical and emotional maltreatment behaviors (N = 5799)

(a) Any physical maltreatment behaviors		
	Partner reported	
	No	Yes
Mother reported		
No	5420 93.5%	151 2.6%
Yes	182 3.1%	46 0.8%

(b) Any emotional maltreatment behaviors		
	Partner reported	
	No	Yes
Mother reported		
No	4891 84.3%	328 5.7%
Yes	418 7.2%	162 2.8%

(c) Agreement between reporters		
Type of maltreatment behavior	ĸ	PABAK^a^
Physical	0.19	0.89^b^
Emotional	0.23	0.74^b^

In panels a and b, cell entries are subsample size and the percentage of entire sample the cell represents. The diagonals show the raw-agreement and the off-diagonals show the raw-disagreement

^a^Prevalence-adjusted bias-adjusted κ

^b^PABAK greater than 0.60 indicates substantial agreement beyond chance

Children retrospectively reported more maltreatment than their caregivers (Table 2). Pair-wise disagreement was more common when comparing children and their caregivers. In 20.4% of pairs (N = 484) the child retrospectively reported PM, but not the caregivers; 4.6% of caregivers (N = 109) reported PM, but not the child; in 27.8% of pairs (N = 659), the child retrospectively reported EM, but not the caregivers; and in 8.9% of pairs (N = 210) the caregivers reported EM, but not the child. Compared to reports between mother–partner pairs, caregivers–child pairs had lower levels of agreement across both behaviors: PM (κ = 0.05; PABAK = 0.50); EM (κ = 0.08; PABAK = 0.27, Table 2).

**Table 2:** Prevalence and agreement between prospective caregiver (combining mother and partner) reports and retrospective child reports on child’s exposure to physical and emotional maltreatment behaviors (N = 2373)

(a) Any physical maltreatment behaviors		
	Caregiver reported	
	No	Yes
Child reported		
No	1726 72.7%	109 4.6%
Yes	484 20.4%	54 2.3%

(b) Any emotional maltreatment behaviors		
	Caregiver reported	
	No	Yes
Child reported		
No	1333 56.2%	210 8.9%
Yes	659 27.8%	171 7.2%

(c) Agreement between reporters		
Type of maltreatment behavior	κ	PABAK^a^
Physical	0.05	0.50^b^
Emotional	0.08	0.27^c^

In panels a and b, caregiver-reported maltreatment was considered “Yes” when maltreatment was reported by mother and/or partner

^a^Prevalence-adjusted bias-adjusted κ

^b^PABAK greater than 0.40 indicates moderate agreement beyond chance

^c^PABAK greater than 0.20 and less than 0.40 indicates fair agreement beyond chance

### Predictors of Disagreement

**Fig. 1:**
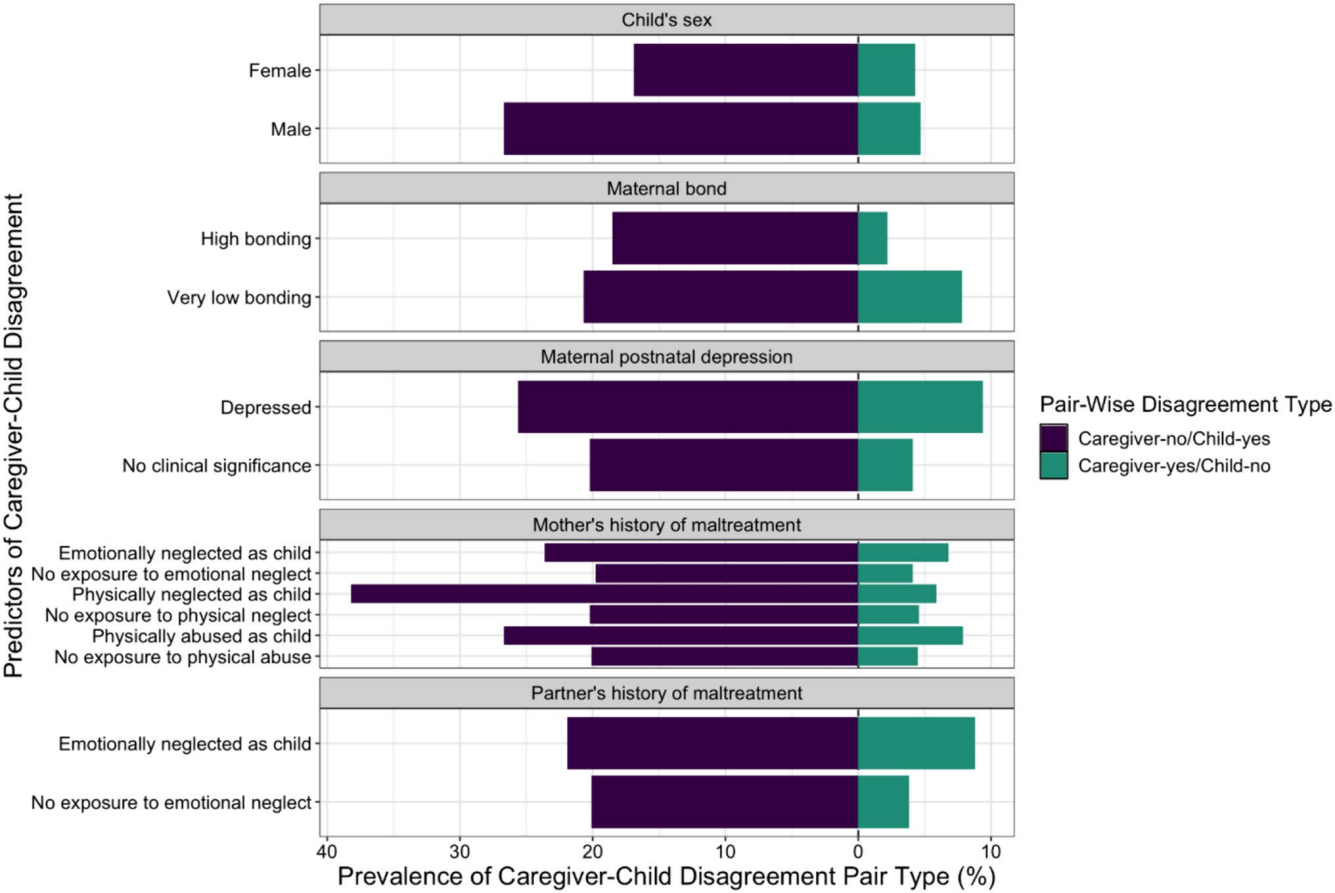
Significant predictors of pair-wise disagreement between prospective caregiver reports and retrospective child reports on child’s exposure to physical maltreatment (N = 2373)

Given the high concordance of mother–partner reports, only results related to predictors of caregiver–child disagreement are presented. Of 16 variables tested, eight were associated with disagreement between reporters for PM (Table 3). Male sex [odds ratio (OR) = 1.69, 95% CI = 1.40–2.04], certain maternal education levels lower than a college degree (less than O-level: OR = 1.46, 95% CI = 1.07–1.99; A-level: OR = 1.48, 95% CI = 1.13–1.93), maternal postnatal depression (OR = 1.69, 95% CI = 1.20–2.37), caregiver history of childhood maltreatment (e.g., mother emotionally neglected: OR = 1.40, 95% CI = 1.11–1.76; mother physically neglected: OR = 2.40, 95% CI = 1.21–4.75; mother physically abused: OR = 1.56, 95% CI = 1.02–2.38; and partner emotionally neglected: OR = 1.42, 95% CI = 1.11–1.83), and lower scores on maternal bonding measurements (OR = 1.53, 95% CI = 1.18–1.98) were significantly associated with reporter PM disagreement, with caregivers tending to underreport PM exposure (Fig. 1). All scores less than the 4th quartile on maternal bonding measurements predicted caregiver–child EM exposure disagreement (1st quartile: OR = 1.28, 95% CI = 1.02–1.62; 2nd quartile: OR = 1.41, 95% CI = 1.10–1.82; 3rd quartile: OR = 1.28, 95% CI = 1.01–1.63).

**Table 3:** Predictors of disagreement between prospective caregiver reports and retrospective child reports on child’s exposure to physical (PM) and emotional (EM) maltreatment (N = 2373)

Factors	Disagreement in PM reporting			Disagreement in EM reporting		
	OR	*p*-value	95% CI	OR	*p*-value	95% CI
Child’s sex						
Female	Ref					
Male	**1.69**	** < 0.01**^**a**^	**[1.40, 2.04]**	1.02	0.80	[0.86, 1.22]
Child’s race						
White	Ref					
Non-white	1.32	0.35	[0.74, 2.35]	1.31	0.32	[0.77, 2.24]
Maternal education						
College degree or above	Ref					
A-level	**1.48**	** < 0.01**	**[1.13, 1.93]**	1.13	0.30	[0.89, 1.44]
O-level	1.17	0.24	[0.90, 1.53]	1.15	0.22	[0.92, 1.45]
Less than O-level	**1.46**	**0.02**	**[1.07, 1.99]**	1.22	0.15	[0.93, 1.62]
Marital status						
Married	Ref					
Never married	0.81	0.44	[0.48, 1.38]	1.00	1.00	[0.60, 1.66]
Widowed/divorced/separated	**0.63**	**0.04**	**[0.40, 0.98]**	0.89	0.60	[0.58, 1.37]
Maternal age						
Ages > 35	Ref					
Ages 20–35	0.89	0.46	[0.65, 1.22]	0.80	0.12	[0.60, 1.06]
Ages 15–19	2.48	0.05	[1.00, 6.14]	2.32	0.07	[0.92, 5.82]
Previous pregnancies						
0	Ref					
1	1.00	0.97	[0.81, 1.24]	0.97	0.77	[0.81, 1.17]
2	0.85	0.30	[0.62, 1.16]	1.05	0.72	[0.80, 1.38]
3 +	1.44	0.13	[0.90, 2.30]	1.07	0.78	[0.68, 1.67]
Maternal postnatal depression^b^						
No clinical significance	Ref					
Depressed	**1.69**	** < 0.01**^**a**^	**[1.20, 2.37]**	0.94	0.70	[0.67, 1.31]
Mother’s history of maltreatment^c^						
Emotionally neglected as child	**1.40**	** < 0.01**	**[1.11, 1.76]**	1.15	0.21	[0.93, 1.42]
Physically neglected as child	**2.40**	**0.01**	**[1.21, 4.75]**	1.06	0.86	[0.53, 2.14]
Physically abused as child	**1.56**	**0.04**	**[1.02, 2.38]**	1.47	0.06	[0.98, 2.19]
Partner’s history of maltreatment^c^						
Emotionally neglected as child	**1.42**	** < 0.01**	**[1.11, 1.83]**	1.10	0.41	[0.87, 1.40]
Physically neglected as child	1.70	0.11	[0.88, 3.30]	1.10	0.77	[0.57, 2.11]
Physically abused as child	1.52	0.05	[1.00, 2.32]	1.45	0.07	[0.97, 2.16]
Maternal mental health history^d^						
History of severe depression	1.21	0.31	[0.84, 1.76]	1.09	0.63	[0.77, 1.53]
Maternal bonding^e^						
Quartile 4	Ref					
Quartile 3	1.27	0.09	[0.96, 1.66]	**1.28**	**0.04**	**[1.01, 1.63]**
Quartile 2	1.30	0.07	[0.98, 1.73]	**1.41**	** < 0.01**	**[1.10, 1.82]**
Quartile 1	**1.53**	** < 0.01**	**[1.18, 1.98]**	**1.28**	**0.04**	**[1.02, 1.62]**
Paternal bonding^e^						
Quartile 4	Ref					
Quartile 3	1.00	0.98	[0.76, 1.33]	1.06	0.65	[0.83, 1.36]
Quartile 2	1.09	0.61	[0.79, 1.50]	1.09	0.55	[0.82, 1.46]
Quartile 1	1.14	0.38	[0.85, 1.53]	1.05	0.72	[0.81, 1.37]

Bold values are significant at p < 0.05

^a^These results remained statistically significant, after controlling for multiple testing using the Bonferroni correction

^b^Maternal depression at 8 months postpartum was evaluated based on total scores from the Edinburgh Postnatal Depression Scale (EPDS); consistent with prior studies [41], scores greater than 12 was indicative of significant depressive symptoms

^c^Referent for these analyses is no exposure to maltreatment (neglect, abuse)

^d^Referent for these analyses is no history. Maternal mental health history of alcoholism and drug addiction are not included in this table due to low prevalence of these conditions in the sample (< 5%)

^e^We transformed maternal and partner bonding scores into categories based on quartiles

### Consequences of Disagreement

Caregiver–child report disagreement on the child’s exposure to maltreatment was associated with a *decreased* risk of several health outcomes. Both PM and EM reporter disagreement was associated with a decreased risk of child’s self-reported history of lifetime depression (PM: OR = 0.60, 95% CI = 0.48–0.74; EM: OR = 0.64, 95% CI = 0.52–0.79) and clinically-assessed generalized anxiety disorder (PM: OR = 0.59, 95% CI = 0.42–0.84; EM: OR = 0.59, 95% CI = 0.42–0.82, Table 4). Reporter disagreement on EM exposure, but not PM exposure, was associated with a statistically significant decreased risk of other lifetime medical conditions (OR = 0.67, 95% CI = 0.54–0.83).

**Table 4:** Estimated associations of child-caregiver disagreement for reports of physical (PM) and emotional (EM) maltreatment on medical, mood, and behavioral health outcomes in the secondary sample (N = 2373)

(A) Medical outcomes						
	Disagreement in PM reporting			Disagreement in EM reporting		
	OR	*p*-value	95% CI	OR	*p*-value	95% CI
Lifetime history of asthma	0.82	0.06	[0.66, 1.01]	0.84	0.08	[0.69, 1.02]
Other lifetime medical condition	0.80	0.06	[0.63, 1.01]	**0.67**	**0.0002**^a^	**[0.54, 0.83]**

	β	*p*-value	95% CI	β	*p*-value	95% CI
General health rating	**0.13**	**0.001**	**[0.06, 0.21]**	**0.16**	** < 0.0001**^a^	**[0.09, 0.23]**
BMI^b^	**0.55**	**0.04**	**[0.01, 1.10]**	0.24	0.32	[− 0.24, 0.73]
Seated systolic blood pressure^b^	1.29	0.05	[0.00, 2.57]	0.33	0.57	[− 0.82, 1.48]
Standing systolic blood pressure^b^	− 1.92	0.11	[− 4.27, 0.43]	− 0.63	0.55	[− 2.70, 1.44]
Seated diastolic blood pressure^b^	0.50	0.26	[− 0.38, 1.39]	0.11	0.78	[− 0.68, 0.90]
Standing diastolic blood pressure^b^	− 1.10	0.19	[− 2.75, 0.54]	− 0.88	0.23	[− 2.33, 0.57]

(B) Mood and behavioral outcomes						
	Disagreement in PM reporting			Disagreement in EM reporting		
	OR	*p*-value	95% CI	OR	*p*-value	95% CI
Lifetime history of depression	**0.60**	** < 0.0001**^a^	**[0.48, 0.74]**	**0.64**	** < 0.0001**^a^	**[0.52, 0.79]**
Current mild depressive episode^b^	**0.46**	** < 0.0001**^a^	**[0.33, 0.64]**	**0.63**	**0.004**	**[0.46, 0.86]**
Current moderate depressive episode^b^	**0.41**	** < 0.0001**^a^	**[0.28, 0.59]**	**0.60**	**0.005**	**[0.42, 0.86]**
Current generalized anxiety disorder^b^	**0.59**	**0.004**	**[0.42, 0.84]**	**0.59**	**0.002**	**[0.42, 0.82]**

	β	*p*-value	95% CI	β	*p*-value	95% CI
Depressive symptom total score^c^	**2.03**	** < 0.0001**^a^	**[1.53, 2.53]**	**1.89**	** < 0.0001**^a^	**[1.44, 2.34]**
Alcohol abuse severity^b^	**0.11**	** < 0.0001**^a^	**[0.06, 0.16]**	**0.10**	** < 0.0001**^a^	**[0.06, 0.15]**
Cannabis abuse severity^b^	**0.23**	** < 0.0001**^a^	**[0.15, 0.32]**	**0.12**	**0.002**	**[0.04, 0.20]**
Nicotine dependence severity^b^	**0.12**	** < 0.0001**^a^	**[0.07, 0.17]**	**0.10**	** < 0.0001**^a^	**[0.05, 0.14]**
Lifetime illicit drug use^b^	**0.40**	** < 0.0001**^a^	**[0.25, 0.56]**	**0.34**	** < 0.0001**^a^	**[0.19, 0.48]**

OR can be interpreted as the increase (or decrease) in odds of the health outcome in the group with caregiver–child disagreement versus caregiver–child agreement in maltreatment reporting. Bold values are significant at p < 0.05

*CI* confidence interval, *OR* odds ratio, *BMI* body mass index

^a^These results remained statistically significant, after controlling for multiple testing using the Bonferroni correction

^b^Outcome was evaluated at the clinic-based assessment at 24 years

^d^Depressive symptoms assessed with the Short Mood and Feelings Questionnaire (SMFQ), self-reported at 22 years

In contrast, caregiver–child disagreement for both types of maltreatment were significantly positively associated with higher ratings on clinical assessments of alcohol abuse, cannabis abuse, nicotine dependence, and lifetime illicit drug use (Table 4). Caregiver–child disagreement for both types of maltreatment was associated with higher ratings of self-reported current depressive symptoms (PM: β = 2.03, 95% CI = 1.53–2.53; EM: β = 1.89, 95% CI = 1.44–2.34). Caregiver–child disagreement on PM exposure was associated with higher BMI, but disagreement on either maltreatment type was otherwise not significantly associated with clinically assessed BMI or blood pressure measures.

Retrospective, but not prospective, reports of PM and EM exposure were significantly associated with increased risk of most health outcomes (Fig. S3).

### Sensitivity Analysis

Fewer maltreatment cases were identified when using a stricter cut-point to define maltreatment (Table S5); PABAK measures of agreement, but not κ measures, improved (Table S6), and associations between disagreement and child health outcomes were generally the same but fewer results were statistically significant when maltreatment frequency was high (often/very often) (Fig. S2).

## Discussion

This study examined the predictors of disagreement between different reporters of child maltreatment exposure and explored implications of reporter disagreement. Like other studies  [33–35], we found that parent and child reports often disagree on maltreatment exposure; however, our results are comparable to the literature only when understanding our data based on PABAK values. Our κ statistics were even lower, thus indicating worse agreement, than a prior meta-analysis (who reported κ = 0.19) [15]. In our sample, maltreatment exposure was rare; rare events contribute to the reliability paradox described earlier, where κ coefficient show poor agreement, but the PABAK value indicated moderate to substantial agreement between reporters. In these instances, PABAK provides a clearer picture of agreement between reporters. Our results may reflect a higher percentage of children never exposed to maltreatment, causing our κ value to be artificially decreased. Considering these findings, future studies should include multiple methods for assessing agreement (κ and PABAK) to account for the possible impacts of sample size and prevalence.

We identified correlates of self- and parent-reported maltreatment experiences of children that predicted reporting disagreement, and these associations provide actionable new insights to improve maltreatment measurement and the identification of high-risk children. While prior studies suggest maternal postnatal depression and caregivers’ own history of maltreatment may increase risk of early-life child maltreatment, our findings suggest these factors may also influence reporting of maltreatment [36, 37]. Caregivers’ social desirability or changes of societal definitions of maltreatment over generations may explain these results, exacerbating potential disagreement between multi-generational reporters. Future studies may adjust for possible underreporting by caregivers of their child’s exposure to maltreatment based on these characteristics. Furthermore, screening measures for childhood maltreatment, especially those with caregiver reporters, may also want to evaluate these characteristics to identify high-risk children. In future studies of child maltreatment, the role of disagreement in both retrospective and prospective reports of children’s maltreatment experiences should be considered.

Consistent with existing literature, we found that retrospective reports were more strongly associated with health outcomes than prospective reports [16]. In our analysis, disagreement between reporters was associated with a *decreased* risk of lifetime depression, anxiety, and poor general health. A possible explanation for why disagreement was associated with decreased risk could be that caregivers may be less likely to acknowledge health problems in their child, thus decreasing the chances their child received healthcare or a diagnosis when showing signs of distress. Another possible explanation is that the typical age of onset for common mental health disorders is after age 22 to 24 when health outcomes were assessed in the ALSPAC study, and as such, studying the mental health consequences of maltreatment reporting disagreement may require longer-term follow-up [38]. Disagreement in maltreatment reports was more common in our analysis among male children, who are less likely to exhibit internalizing symptoms in response to trauma than their female counterparts and more likely to express externalizing symptoms and substance use behaviors [39].

Although disagreement in reporting was associated with decreased risk for certain mental health diagnoses and overall poor health, we also observed that disagreement was associated with higher self-reported and clinically assessed health problems, including elevated self-reported depressive symptoms, substance use severity, and BMI. Given the association of disagreement to depression symptoms, but not a depression or anxiety diagnosis, disagreement may be a risk factor for unmet mental health need or future mental health risk. Findings may also reflect sex differences in behavioral symptoms among children who experience maltreatment [39].

### Limitations

Several limitations should be considered when interpreting these results. First, our study examines maltreatment exposure before age 11, thus our results may not be generalizable to maltreatment occurring in later childhood and adolescence. Second, differences in maltreatment survey depth could have biased our results towards increased disagreement. Although mothers and partners identified the perpetrator of maltreatment, our definition of agreement was not based on the identity of the perpetrator. Therefore, a mother and partner pair could be coded as in agreement on the child’s exposure to maltreatment but have disagreement on *who* was responsible. Likewise, disagreement between caregiver–child pairs could be due to the child being maltreated by another adult in the family, although perpetrators of the maltreatment are most often a child’s parent [40]. Finally, there are several types of bias including memory, recall, and reporting bias that may affect how ALSPAC participants responded to surveys. Future studies should focus on the way maltreatment is screened and how the identity of the perpetrator of maltreatment influences multi-informant disagreement.

### Summary

Our study found that retrospective child reports and prospective caregiver reports of child maltreatment often disagree, and caution should be taken in using these reports interchangeably. We identified predictors of disagreement in reporting including child male sex, maternal education levels lower than a college degree, maternal postnatal depression, caregiver history of childhood maltreatment, and impaired maternal bonding. Further research is needed to understand what factors drive disagreement and how to optimize child maltreatment assessment in clinical practice accounting for the perspectives of both caregivers and children themselves. Disagreement between reporters may be important to consider when exploring the mechanisms underlying the connection between child maltreatment, poor health outcomes, and type of report, as well as possible unmet need for mental health evaluation.

## Supplementary Information

Below is the link to the electronic supplementary material.

Supplementary file1 (DOCX 850 kb)

## Data Availability

ALSPAC data may be requested for use in research at the following link: https://www.bristol.ac.uk/alspac/researchers/access/.
