# Supplementary material for: The Prevalence, Predictors, and Health Consequences of Disagreement in Reports of Child Maltreatment Exposure
Source: Child Psychiatry Hum Dev. 2024 May 30;57(2):404–14. doi: 10.1007/s10578-024-01721-2 (PMC13128786; doi:10.1007/s10578-024-01721-2)
Supplement: Supplementary file 1 — Supplementary file1 (DOCX 850 kb) [file 10578_2024_1721_MOESM1_ESM.docx]

SUPPLEMENTAL MATERIALS

**Section 1: Sample Description**

Data came from the Avon Longitudinal Study of Parents and Children (ALSPAC), an ongoing longitudinal birth cohort from Avon, England.^1^ ALSPAC was designed to increase knowledge of the pathways to health across the lifespan, with an emphasis on genetic and environmental determinants. The initial ALSPAC sample consisted of 14,541 pregnancies and 14,676 fetuses; further recruitment when the oldest children were 7 years old increased this cohort to 15,454 pregnancies and 15,589 fetuses, resulting in 14,901 children alive at 1 year of age. Informed consent for the use of data collected via questionnaires and clinics was obtained from participants following the recommendations of the ALSPAC Ethics and Law Committee at the time. ALSPAC has collected data on genetic, biological, psychological, and other exposures in relation to a wide range of health and social outcomes.^1-4^ The ALSPAC website contains more details of the data: <http://www.bristol.ac.uk/alspac/researchers/our-data/>.

Primary and secondary analytic samples

Starting with the total ALSPAC analytic sample of singleton births (n=15,240), we removed participants where the mother and/or the partner did not complete any maltreatment-related questionnaires at timepoints of interest (see below for more details about timepoints explored). We were interested in examining pairs of reporters who answered maltreatment-related questions, so eliminating those without any data was necessary to explore reporter agreement. From this work, we obtained a base sample of n=8784. From this base, we created 2 analytic subsamples (**Figure S1**).

Our first analytic subsample, labeled “primary sample”, was used to examine agreement between mother and partner reports. Mother and partners prospectively reported on their own and each other’s physical and emotional maltreatment behaviors towards their child. Examination of these parental reports from two different reporters across multiple timepoints in the child’s life allows for an increased chance that we are accurately capturing those who experienced childhood maltreatment. There were 7 time points where maltreatment items were evaluated by both mother and partner report, specifically when the child was at the age of: 8 months, 1 year and 9 months, 2 years and 9 months, 4 years and 11 months, 5 years and 1 month, 6 years, and 9 years. The distribution of survey completion rates can be seen in **Supplemental Table 1**. The primary sample included participants who had 3 or more completed questionnaires from any of the 7 timepoints of interest regarding child maltreatment behaviors for mother reports *and* who had 3 or more completed questionnaires from any of the 7 timepoints of interest regarding child maltreatment behaviors for partner reports (N=5799 pairs).

Our “secondary sample” was restricted to include only the participants from the primary analytic sample who had retrospective child-reported data for maltreatment questions (**Figure S1**); this sample allowed us to examine the differences in reports of maltreatment by caregivers (combined mother and partner reports) and children (N=2373 pairs). Demographic characteristics of these 2 subsamples are shown in **Supplemental Table 2**.

**Section 2: Maltreatment Exposure Measures**

We examined 2 types of child maltreatment measured using reports from mail-in questionnaires, which were sent to mothers and partners. Data from young-adult children was collected and managed using REDCap electronic data capture tools hosted at the University of Bristol. REDCap (Research Electronic Data Capture) is a secure, web-based software platform designed to support data capture for research studies^5^. Participants were informed that all their responses were confidential.

Caregiver-reported maltreatment behaviors*.* In the primary sample comparing mother and partner reports, maltreatment behaviors were coded as affirmative responses to any of the following items by the mother and partner separately: (a) Your partner was physically cruel to your children; (b) You were physically cruel to your children; (c) Your partner was emotionally cruel to your children; and (d) You were emotionally cruel to your children. In the secondary sample, a “caregiver” report was created in which physical and emotional maltreatment behaviors, respectively, were coded if the mother, partner, or both parties responded affirmatively to any of the following items: (a) Your partner was physically cruel to your children; (b) You were physically cruel to your children; (c) Your partner was emotionally cruel to your children; and (d) You were emotionally cruel to your children.

Child-reported experiences of maltreatment*.* Children retrospectively reported on 8 negative experiences that could have occurred before age 11, where the perpetrator was an adult in the family, using a 5-point frequency scale: never, rarely, sometimes, often, or very often. The negative experience items were based on the validated Child Abuse Questionnaire and the Sexual Experiences Survey.^6,7^ There were 3 items excluded from the analysis to concentrate on maltreatment behaviors rather than the broader category of negative family experiences, including 2 items related to corporal punishment: (1) Adult in family punished you in a way that seemed cruel (endorsed by 16.7% of secondary sample); and (2) Adult in family smacked you for discipline (endorsed by 62.1% of secondary sample). The third item was excluded due to it being highly endorsed by the secondary sample (93.8%) which could have skewed our results to overreport maltreatment exposure: (3) Adult in family shouted at you.

Child-reported experiences: Any frequency of maltreatment. We examined 5 maltreatment behaviors reported retrospectively by adult-aged children. Physical maltreatment items included: (1) Adult in family pushed, grabbed or shoved you; (2) Adult in family kicked, punched, hit, or physically attacked you; and (3) Adult in family hit you so hard it left bruises or marks. Emotional maltreatment items included: (1) Adult in family threatened to kick, punch, hit, or physically attack you; and (2) Adult in family said hurtful or insulting things to you. Because child maltreatment is commonly underreported (rather than overreported),^8,9^ children were coded as exposed if they reported any frequency of maltreatment greater than “never”, consistent with prior studies.^10^ However, we also performed sensitivity analyses, as described later.

Child-reported experiences: Moderate or high frequency of maltreatment. Recognizing there is no standard way to define maltreatment, in a set of sensitivity analyses we examined the prevalence of exposure to maltreatment behaviors (**Table S5**) and agreement between prospective caregiver reports and retrospective child reports (**Table S6**) using a stricter definition of exposure. Specifically, we defined child maltreatment exposure as: (a) *Any Frequency*: Child reported at least one maltreatment item at a frequency of rare, sometimes, often or very often; (b) *Moderate Frequency*: Child reported at least one maltreatment item at a frequency of sometimes, often or very often; and (c) *High Frequency*: Child reported at least one maltreatment item at a frequency of often or very often.

**Section 3: Predictors of Disagreement**

As described, we performed logistic regression analyses to compare the associations of social, demographic, and familial factors as predictors of discordance between: (a) mother and partner reports in the primary sample and (b) caregiver and child reports in the secondary sample. Below we provide more specific details regarding covariate measurement not already covered in the main text.

Maternal postnatal depression was self-reported by mothers at 8 months postpartum with the Edinburgh Postnatal Depression Scale (EPDS).^11^ A cut-off score of 13 or higher determined that maternal depression was present, as recommended in the literature.^11,12^

Additionally, the mother’s self-reported lifetime history of severe depression, addiction, and alcoholism was also examined using data provided by maternal survey at 12 weeks’ gestation. Mothers were asked to report on 24 health conditions and behaviors via the following responses: (A) Yes, had it recently, (B) Yes in past, not now, (C) No, never, and (D) Don’t know. A response of yes, whether recently or in the past, was used to classify mothers as having a lifetime history of severe depression, addiction, and alcoholism.

History of childhood maltreatment for the mothers and partners was also assessed. Mothers and partners reported on their childhood experiences of emotional neglect, physical neglect, and physical abuse at the 33-month report. Participants were asked to report whether they had been exposed to emotional neglect, physical neglect, and physical abuse during their childhood using the following responses: (A) Yes, severely neglected/abused; (B) Yes, somewhat neglected/abused; and (C) No, not at all. Responses were recoded such that any endorsement of maltreatment (i.e., yes, severely neglected/abused or yes, somewhat neglected/abused) was consider as exposed.

Maternal and paternal bonding to the child were also assessed. Mothers and partners completed a questionnaire concerning bonding at the 8 month report. Participants were asked to rate how applicable statements concerning bonding with their baby (i.e., “I really enjoy my baby”). A total “bonding score” (range 4-44) was derived with higher scores indicating closer bonding to the child.^13^ For our analyses, we transformed the bonding scores into categories based on quartiles of the dataset.

For these analyses, we used a dichotomized outcome variable (concordant versus discordant) for emotional and physical maltreatment behaviors presence.

**Section 4: Health Consequences of Disagreement**

Data on medical, mood, and behavioral health outcomes in adult-aged children came from 2 sources: (A) a self-report mail-in questionnaire given to children at age 22 years and (B) an interviewer-based clinic-based assessment of the children at age 24 years. Given that self-reported measures of health may understate disease burden or severity,^14,15^ we wanted to explore both self-reported and clinic-based data. For the reporting of our results, we split health consequences into two groups: (1) medical outcomes and (2) mood and behavioral outcomes. Health conditions were only explored further if they were present in at least 5% of the secondary analytic sample.

Medical Outcomes. From the 22-year self-report, we collected information on the following. First, participants reported on their lifetime history of the following health outcomes: asthma, hypertension, heart attack/myocardial infarction, stroke, Type 1 diabetes, Type 2 diabetes, chronic fatigue, and other medical condition(s). Participants were asked if they had ever been diagnosed with any of these health conditions and via the following responses: (A) Yes, by doctor, (B) Yes, by self, (C) No, and (D) Not sure. Participants were coded as exposed to a health condition if they were diagnosed by a doctor or by themselves. Second, participants were asked about self-reported general health quality (on a scale of increasingly worse health from: 1=excellent, 2=very good, 3=good, 4=fair, or 5=poor). We treated health quality as a continuous variable for our analyses. Finally, clinically evaluated measures of physical health at the 24-year timepoint were explored including: Body Mass Index (BMI; calculated as weight in kilograms divided by height in meters squared), seated and standing systolic blood pressure, and seated and standing diastolic blood pressure.

Mood and Behavioral Outcomes. From the 22-year self-report, we collected information on the following. Participants reported on their lifetime history of the following behavioral health outcomes: depression, bipolar disorder, and schizophrenia. Participants were asked if they had ever been diagnosed with any of these health conditions and via the following responses: (A) Yes, by doctor, (B) Yes, by self, (C) No, and (D) Not sure. Participants were coded as exposed to a health condition if they were diagnosed by a doctor or by themselves. Participants were also asked whether they had had a suicide attempt since age 21 and how much this impacted them. Participants were offered the following responses: (A) Yes, affected me a lot, (B) Yes, moderately affected, (C) Yes, mildly affected, (D) Yes, but didn’t affect me at all, and (E) No, did not happen. Any responses where the child indicated a suicide attempt since age 21 was coded as being exposed to this health behavior. At this same 22-year time-point, participants also completed the Short Mood and Feelings Questionnaire^16^ which is a self-report, 13-question screener for depressive symptoms over the preceding 2 weeks. Higher scores are indicative of more severe depressive symptoms.

We examined current mood and anxiety disorders as evaluated clinically by the Computerized Interview Schedule – Revised (CIS-R)^17^ at the 24-year clinic-based assessment. The CIS-R is a self-administered computerized interview that derives diagnoses based on ICD-10 criteria. Specifically, we examined the presence/absence of the following disorders as assessed by the CIS-R: major depressive episode (mild, moderate, severe), generalized anxiety disorder, social phobia disorder, specific phobia disorder, and panic disorder.

We also explored health outcomes assessed at the 24-year clinic-based assessment related to alcohol, cannabis, nicotine, and other illicit drug use. Alcohol use disorder (AUD) was measured on criteria set by Diagnostic and Statistical Manual of Mental Disorders V (DSM-5)^18^ (on a severity scale: 0=none, 1=mild AUD, 2=moderate AUD, and 3=severe AUD). Cannabis use and abuse were measured by the Cannabis Abuse Screening Test (CAST)^19^ wherein, for individuals who used cannabis in the past 12 months, a raw score of 0 to 6 was derived. In our analysis, we created a severity rating such that: 0=never tried cannabis, 1=no cannabis in the past 12 months, 2=CAST score is 0 (has used cannabis in the past 12 months), and 3=CAST score is greater than or equal to one (has used cannabis in the past 12 months). Nicotine dependence was measured via the Fagerström Test for Nicotine Dependence^20^ and investigated based on a severity scale (0=no dependence, 1=very low dependence, 2=low dependence, 3=medium dependence, 4=high dependence, and 5=very high dependence). The total number of different illicit drugs across lifetime was assessed and treated as a continuous variable for our analyses.

**Figure S1. Construction of the two analytic samples.**

| **Original Total Sample** ^a^ : N = 15,240 | |
| --- | --- |
|  |  |
| **Base Sample**: Remove child participants where the mother and/or the partner did not complete any maltreatment-related questionnaires at time points of interest ^b^.  N = 8784 | |
|  |  |
| **Primary Sample**: Restrict base sample to include mother-partner pairs with 3 or more completed maltreatment exposure questionnaires at time points of interest by both mother and partner ^b^.  N = 5799 | |
|  |  |
| **Secondary Sample**: Exclude those without data for the child retrospective report of child maltreatment exposure.  N = 2373 | |
| ^a^*After additional wave of recruitment when the oldest children were 7 years old, the sample increased to 15,589 fetuses. We included only singletons in our original total sample, giving us an N of 15,240.*  *^b^ See Table S3 for further details about timepoints assessed, which included: 8 months, 1.75 years, 2.75 years, 4 years, 5 years, 6 years, and 9 years.* | |

| **Table S1. Distribution of the total number of time points with data on maltreatment behaviors, out of the 7 time points examined, for mother and partner reports in the original sample (N=15240) and the primary sample (N=5799).** | | | | |
| --- | --- | --- | --- | --- |
|  | Original Total Sample (%, n) | | Primary Sample (%, n) | |
| Total number of time-points with maltreatment behavior data | Mother | Partner | Mother | Partner |
| 0 | 19.6% (2989) | 42.1% (6410) | -- | -- |
| 1 | 7.8% (1182) | 11.6% (1770) | -- | -- |
| 2 | 5.0% (767) | 7.9% (1209) | -- | -- |
| 3 | 4.9% (751) | 6.1% (935) | 2.5% (146) | 15.5% (901) |
| 4 | 6.0% (909) | 5.5% (842) | 5.14% (298) | 14.4% (835) |
| 5 | 7.1% (1086) | 6.1% (923) | 7.5% (435) | 15.8% (918) |
| 6 | 12.3% (1876) | 7.9% (1199) | 16.9% (981) | 20.6% (1195) |
| 7 | 37.3% (5680) | 12.8% (1952) | 67.9% (3939) | 33.6% (1950) |
| Total | 100% (15240) | 100% (15240) | 100% (5799) | 100% (5799) |
| Children retrospectively reported on maltreatment that occurred before age 11. Although mother-partner pairs completed questionnaires with data on maltreatment behaviors at timepoints beyond 11 years, we limited our analyses to roughly match that of the child report, such that we investigated data from mother-partner pairs at 7 timepoints: 8 months, 21 months, 33 months, 47 months, 61 months, 6 years, and 9 years. Like similar longitudinal studies, rates of attrition in ALSPAC are substantial^2,21^ and studies have linked certain characteristics to ongoing participation.^21,22^ The primary sample included mother-partner pairs who had 3 or more completed questionnaires regarding child maltreatment behaviors for mother reports *and* who had 3 or more completed questionnaires regarding child maltreatment behaviors for partner reports. | | | | |

| **Table S2. Comparison of sample demographics of the original sample (N=15240) to primary sample (N=5799), and the original sample (N=15240) to secondary sample (N=2373).** | | | | | | | | | | |
| --- | --- | --- | --- | --- | --- | --- | --- | --- | --- | --- |
|  | |  | **Original Sample** | | **Primary Sample** | | ***P-*value** | **Secondary Sample** | | ***P-*value** |
|  | |  | **N** | **%** | **N** | **%** |  | **N** | **%** |  |
| **Child’s Sex** | | |  |  |  |  | <0.0001 |  |  | <0.0001 |
|  | | Male | 7336 | 51.3 | 2980 | 51.4 |  | 885 | 37.3 |  |
|  | | Female | 6978 | 48.7 | 2819 | 48.6 |  | 1488 | 62.7 |  |
| **Child’s Race** | | |  |  |  |  | <0.0001 |  |  | <0.0001 |
|  | | White | 11190 | 95.0 | 5510 | 97.4 |  | 2269 | 97.6 |  |
|  | | Non-White | 593 | 5.0 | 147 | 2.6 |  | 56 | 2.4 |  |
| **Maternal Education** | | |  |  |  |  | <0.0001 |  |  | <0.0001 |
|  | | Less than O-level | 3641 | 30.0 | 1199 | 21.0 |  | 351 | 15.0 |  |
|  | | O-level | 4191 | 34.6 | 1967 | 34.6 |  | 771 | 32.9 |  |
|  | | A-level | 2729 | 22.5 | 1509 | 26.5 |  | 677 | 28.9 |  |
|  | | College Degree or Above | 1557 | 12.8 | 1016 | 17.9 |  | 543 | 23.2 |  |
| **Maternal Marital Status** | | |  |  |  |  | <0.0001 |  |  | <0.0001 |
|  | | Never Married | 2454 | 19.1 | 613 | 10.7 |  | 202 | 8.6 |  |
|  | | Widowed/Divorced/Separated | 771 | 6.0 | 258 | 4.5 |  | 92 | 3.9 |  |
|  | | Married | 9592 | 74.8 | 4859 | 84.8 |  | 2060 | 87.5 |  |
| **Age of Mother at Child’s Birth** | | |  |  |  |  | <0.0001 |  |  | <0.0001 |
|  | | Ages 15-19 | 648 | 4.8 | 94 | 1.6 |  | 21 | 0.9 |  |
|  | | Ages 20-35 | 12025 | 88.3 | 5224 | 90.1 |  | 2136 | 90.0 |  |
|  | | Ages >35 | 944 | 6.9 | 481 | 8.3 |  | 216 | 9.1 |  |
| **Number of Previous Pregnancies** | | |  |  |  |  | <0.0001 |  |  | <0.0001 |
|  | | None | 5686 | 44.9 | 2728 | 48.0 |  | 1216 | 52.2 |  |
|  | | 1 | 4406 | 34.8 | 1950 | 34.3 |  | 757 | 32.5 |  |
|  | | 2 | 1806 | 14.3 | 756 | 13.3 |  | 271 | 11.6 |  |
|  | | 3+ | 752 | 5.9 | 251 | 4.4 |  | 86 | 3.7 |  |
| **Maternal Depression ^a^** | | |  |  |  |  | <0.0001 |  |  | 0.0004 |
|  | | Not clinically significant depression (≤12) | 10099 | 91.3 | 5265 | 92.9 |  | 2169 | 93.1 |  |
|  | | Depressed (>12) | 962 | 8.7 | 404 | 7.1 |  | 160 | 6.9 |  |
| **Mother emotionally neglected as child** | | |  |  |  |  | 0.09 |  |  | 0.0005 |
|  | | No | 7402 | 78.2 | 4352 | 78.8 |  | 1852 | 80.8 |  |
|  | | Yes | 2063 | 21.8 | 1170 | 21.2 |  | 440 | 19.2 |  |
| **Mother physically neglected as child** | | |  |  |  |  | 0.32 |  |  | 0.05 |
|  | | No | 9289 | 98.0 | 5424 | 98.1 |  | 2259 | 98.5 |  |
|  | | Yes | 188 | 2.0 | 103 | 1.9 |  | 34 | 1.5 |  |
| **Mother physically abused as child** | | |  |  |  |  | 0.0001 |  |  | <0.0001 |
|  | | No | 8856 | 93.8 | 5215 | 94.6 |  | 2187 | 95.6 |  |
|  | | Yes | 589 | 6.2 | 300 | 5.4 |  | 101 | 4.4 |  |
| **Partner emotionally neglected as child** | | |  |  |  |  | 0.01 |  |  | <0.0001 |
|  | | No | 4281 | 80.0 | 3920 | 80.4 |  | 1703 | 82.9 |  |
|  | | Yes | 1069 | 20.0 | 953 | 19.6 |  | 351 | 17.1 |  |
| **Partner physically neglected as child** | | |  |  |  |  | 0.19 |  |  | 0.03 |
|  | | No | 5229 | 97.5 | 4770 | 97.6 |  | 2021 | 98.1 |  |
|  | | Yes | 133 | 2.5 | 117 | 2.4 |  | 39 | 1.9 |  |
| **Partner physically abused as child** | | |  |  |  |  | 0.03 |  |  | 0.06 |
|  | | No | 5050 | 94.2 | 4614 | 94.5 |  | 1956 | 95.0 |  |
|  | | Yes | 309 | 5.8 | 271 | 5.6 |  | 103 | 5.0 |  |
| **Maternal self-reported history of alcoholism** | | |  |  |  |  | 0.02 |  |  | 0.07 |
|  | | No | 12064 | 99.1 | 5654 | 99.3 |  | 2327 | 99.4 |  |
|  | | Yes | 112 | 0.9 | 40 | 0.7 |  | 14 | 0.6 |  |
| **Maternal self-reported history of drug addiction** | | |  |  |  |  | 0.0001 |  |  | 0.08 |
|  | | No | 12117 | 99.5 | 5679 | 99.7 |  | 2335 | 99.7 |  |
|  | | Yes | 59 | 0.5 | 15 | 0.3 |  | 6 | 0.3 |  |
| **Maternal self-reported history of depression** | | |  |  |  |  | <0.0001 |  |  | <0.0001 |
|  | | No | 11082 | 91.0 | 5291 | 92.9 |  | 2193 | 93.7 |  |
|  | | Yes | 1094 | 9.0 | 403 | 7.1 |  | 148 | 6.3 |  |
| **Maternal bonding score** | | |  |  |  |  | 0.0002 |  |  | <0.0001 |
|  | Quartile 1 | | 2734 | 25.4 | 1436 | 25.8 |  | 642 | 28.0 |  |
|  | Quartile 2 | | 3282 | 30.5 | 1775 | 31.9 |  | 751 | 32.7 |  |
|  | Quartile 3 | | 1438 | 13.4 | 731 | 13.2 |  | 302 | 13.2 |  |
|  | Quartile 4 | | 3308 | 30.7 | 1615 | 29.1 |  | 599 | 26.1 |  |
| **Paternal bonding score** | | |  |  |  |  | <0.0001 |  |  | 0.0003 |
|  | Quartile 1 | | 1737 | 25.4 | 1305 | 25.4 |  | 573 | 26.9 |  |
|  | Quartile 2 | | 1901 | 27.8 | 1471 | 28.6 |  | 629 | 30.0 |  |
|  | Quartile 3 | | 1679 | 24.5 | 1292 | 25.1 |  | 516 | 24.2 |  |
|  | Quartile 4 | | 1525 | 22.3 | 1067 | 20.8 |  | 410 | 19.2 |  |
| ^a^ Maternal depression at 8 months postpartum was evaluated based on total scores self-reported by mailed questionnaire from the Edinburgh Postnatal Depression Scale (EPDS); consistent with prior studies,^11^ scores greater than 12 was indicative of significant depressive symptoms. | | | | | | | | | | |

| **Table S3. Summary of the maltreatment measures and the time periods covered by each item for each type of reporter.** | | |
| --- | --- | --- |
| Reporter | Description | Time period covered |
| Mother and Partner | Exposure to maltreatment behaviors determined through ALSPAC-derived mailed questionnaires administered separately to the mother and the mother’s partner. The following items were asked at each of 7 time-points (response options were: yes & affected me a lot, yes moderately affected, yes mildly affected, yes but did not affect me at all, no did not happen; responses were recoded to binary yes/no):  Physical maltreatment items included:  (1) Your partner was physically cruel to your children.  (2) You were physically cruel to your children.  Emotional maltreatment items included:  (1) Your partner was emotionally cruel to your children.  (2) You were emotionally cruel to your children. | Prospective assessment and period examined ^a^:  (1) 8 months (m): birth to 8m  (2) 1.75 years (y): 8m-1.75y  (3) 2.75y: 1.5-2.75y  (4) 4y: 2.5-4y  (5) 5y: 4-5y  (6) 6y: 5-6y  (7) 9y: 6-9y |
| Adult-aged Children | Exposure to maltreatment behaviors by a family member determined through mailed questionnaires administered to children at the age 22 years report and questions were derived from the validated Child Abuse Questionnaire and the Sexual Experiences Survey.^6,7^  Physical maltreatment items included:  (1) Adult in family pushed, grabbed or shoved you.  (2) Adult in family kicked, punched, hit, or physically attacked you.  (3) Adult in family hit you so hard it left bruises or marks.  Emotional maltreatment items included:  (1) Adult in family threatened to kick, punch, hit, or physically attack you.  (2) Adult in family said hurtful or insulting things to you. | Retrospective assessment at 22 years, covering birth to 11years |
| ^a^ At each timepoint, mothers and partners were asked about maltreatment that occurred since the last reporting period or another specified amount of time (i.e. 8 month assessment: maltreatment that may or may not have occurred from child’s birth to 8 months). | | |

| **Table S4. Identity of participant completing partner report by each time point in the primary sample (N=5799) and secondary sample (N=2373).** | | | | |
| --- | --- | --- | --- | --- |
|  | Primary Sample | | Secondary Sample | |
| Timepoint | Child’s Father | Other | Child’s Father | Other |
| 8 months | 98.4% (4989) | 1.6% (80) | 98.8% (2069) | 1.2% (26) |
| 21 months | 92.6% (4676) | 7.4% (374) | 93.5% (1987) | 6.5% (138) |
| 33 months | 97.2% (4624) | 2.8% (133) | 98.5% (1981) | 1.5% (31) |
| 47 months | 96.9% (4414) | 3.1% (141) | 98.0% (1905) | 2.0% (38) |
| 61 months | 96.5% (4056) | 3.5% (147) | 97.3% (1812) | 2.7% (51) |
| 6 years | 92.1% (3713) | 7.9% (320) | 93.2% (1718) | 6.8% (126) |
| 9 years | 95.4% (3121) | 4.6% (149) | 96.2% (1545) | 3.8% (61) |
| Each time point had different response options for the identity of the person completing the “partner” report. The ‘other’ category is a combination of all responses that were not the child’s father, including the mother’s male partner, mother’s female partner, or another person. | | | | |

| **Table S5. Sensitivity Analyses: Retrospective child-reported exposure to maltreatment based on different frequency-related definitions of exposure in the secondary sample (N=2373).** | | | |
| --- | --- | --- | --- |
|  | Exposed (n, %) | | |
| Type | Any Frequency | Moderate Frequency | High Frequency |
| Physical maltreatment | 538 (22.67) | 161 (6.78) | 36 (1.52) |
| Emotional maltreatment | 830 (34.98) | 305 (12.85) | 76 (3.20) |
| Children reported on exposure to maltreatment behavior items based on a 5-item frequency scale where they rated events as occurring: never, rare, sometimes, often, or very often. We created three frequency-based definitions of child-reported exposure with the following categories: (a) *Any Frequency*: Child reported at least 1 maltreatment item at a frequency of rare, sometimes, often or very often; (b) *Moderate Frequency*: Child reported at least 1 maltreatment item at a frequency of sometimes, often or very often; and (c) *High Frequency*: Child reported at least 1 maltreatment item at a frequency of often or very often. | | | |

| **Table S6. Sensitivity analyses: Agreement between caregiver and child reports of parent-to-child maltreatment based on different definitions of child-reported exposure to maltreatment behaviors.** | | | |
| --- | --- | --- | --- |
| Definition of child-reported exposure to maltreatment | Type | ĸ | PABAK^a^ |
| Any Frequency | Physical | 0.05 | 0.50 ^b^ |
|  | Emotional | 0.09 | 0.27 |
|  |  |  |  |
| Moderate Frequency | Physical | 0.08 | 0.77 ^c^ |
|  | Emotional | 0.10 | 0.55 ^b^ |
|  |  |  |  |
| High Frequency | Physical | 0.05 | 0.84 ^c^ |
|  | Emotional | 0.04 | 0.65 ^c^ |
| Children reported on exposure to maltreatment behavior items based on a 5-item frequency scale where they rated events as occurring: never, rare, sometimes, often, or very often. We created three frequency-based definitions of child-reported exposure with the following categories: (a) *Any Frequency*: Child reported at least 1 maltreatment item at a frequency of rare, sometimes, often or very often; (b) *Moderate Frequency*: Child reported at least 1 maltreatment item at a frequency of sometimes, often or very often; and (c) *High Frequency*: Child reported at least 1 maltreatment item at a frequency of often or very often.  ^a^ Prevalence-adjusted bias-adjusted kappa  ^b^ PABAK greater than 0.40 (moderate agreement beyond chance)  ^c^ PABAK greater than 0.60 (substantial agreement beyond chance) | | | |

| **Figure S2. Sensitivity analyses: Estimated associations on medical and mood/behavioral health** **outcomes of child-caregiver disagreement in the secondary sample using different frequency-based definitions of child-reported maltreatment exposure (N=2373).** |
| --- |
| A. Categorical outcomes. |
| **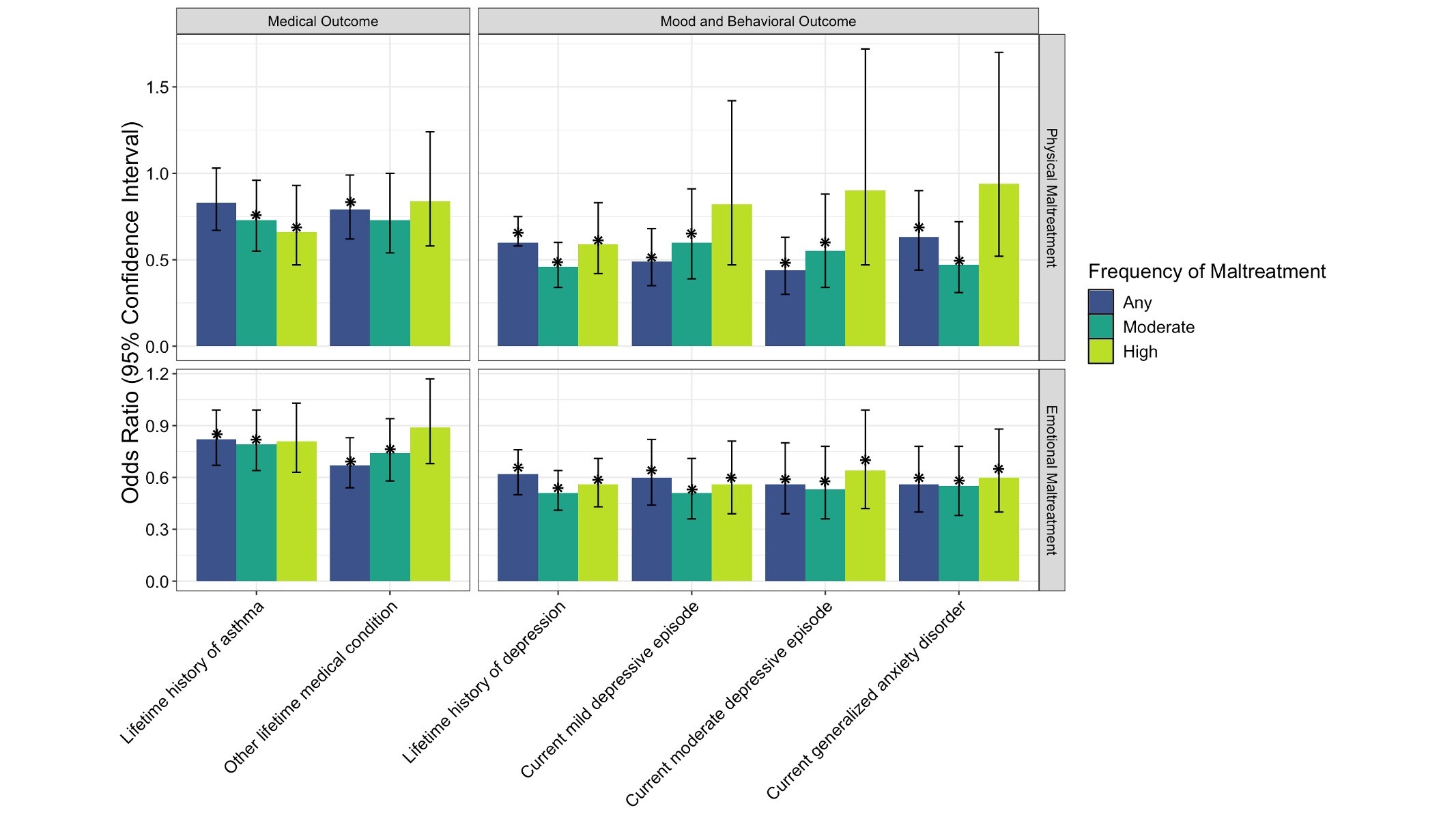** |
| B. Continuous outcomes. |
| **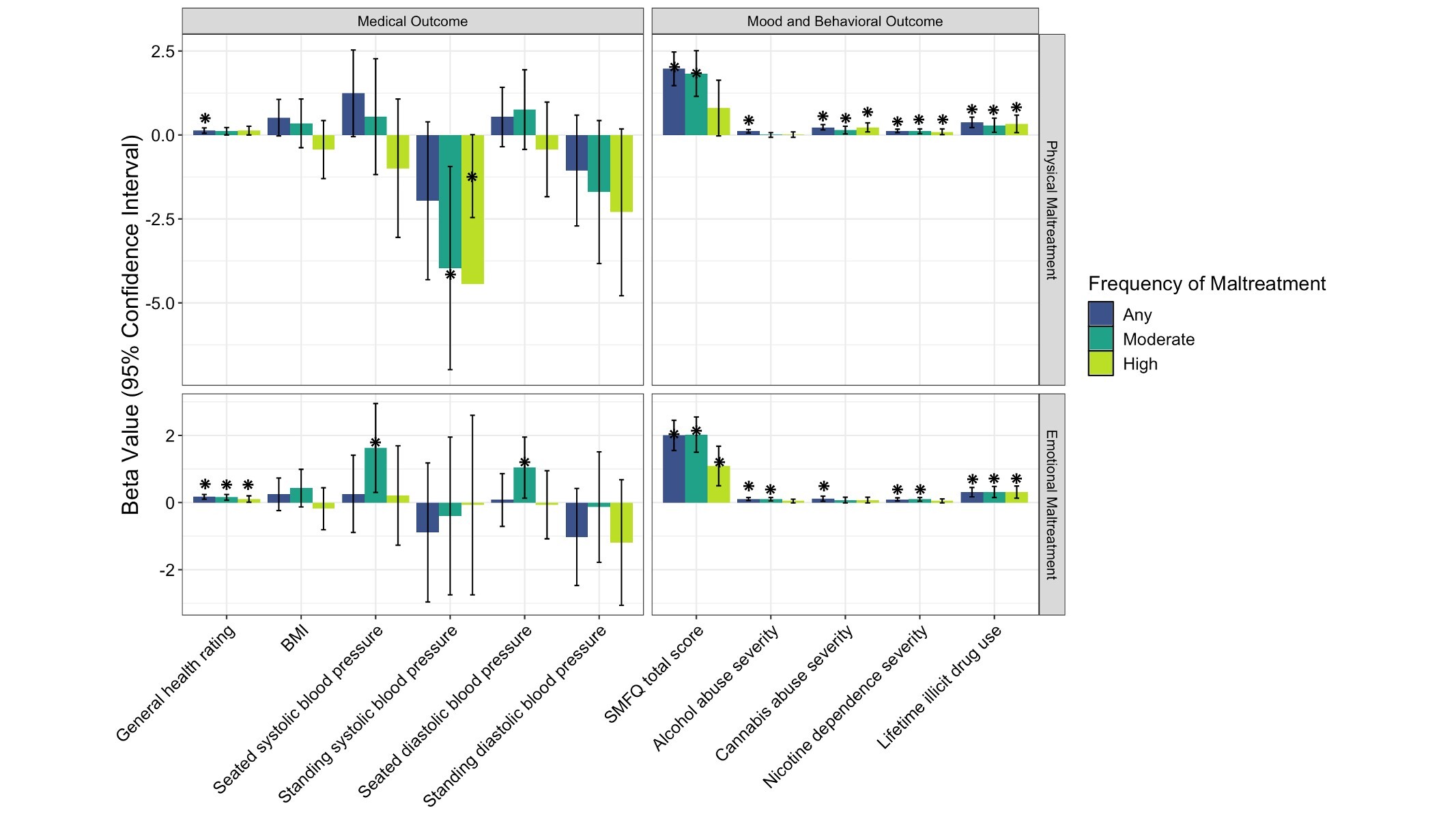** |
| Children reported on exposure to maltreatment behavior items based on a 5-item frequency scale where they rated events as occurring: never, rare, sometimes, often, or very often. We created three frequency-based definitions of child-reported exposure with the following categories: (a) *Any Frequency*: Child reported at least 1 maltreatment item at a frequency of rare, sometimes, often or very often; (b) *Moderate Frequency*: Child reported at least 1 maltreatment item at a frequency of sometimes, often or very often; and (c) *High Frequency*: Child reported at least 1 maltreatment item at a frequency of often or very often.  *Indicates statistically significant results p<0.05. |

| **Figure S3. Secondary analysis: Estimated associations on medical and mood/behavioral health outcomes of retrospective child-reported exposure to maltreatment and prospective caregiver-reported exposure to maltreatment in the secondary sample (N=2373).** |
| --- |
| A. Categorical outcomes. |
| 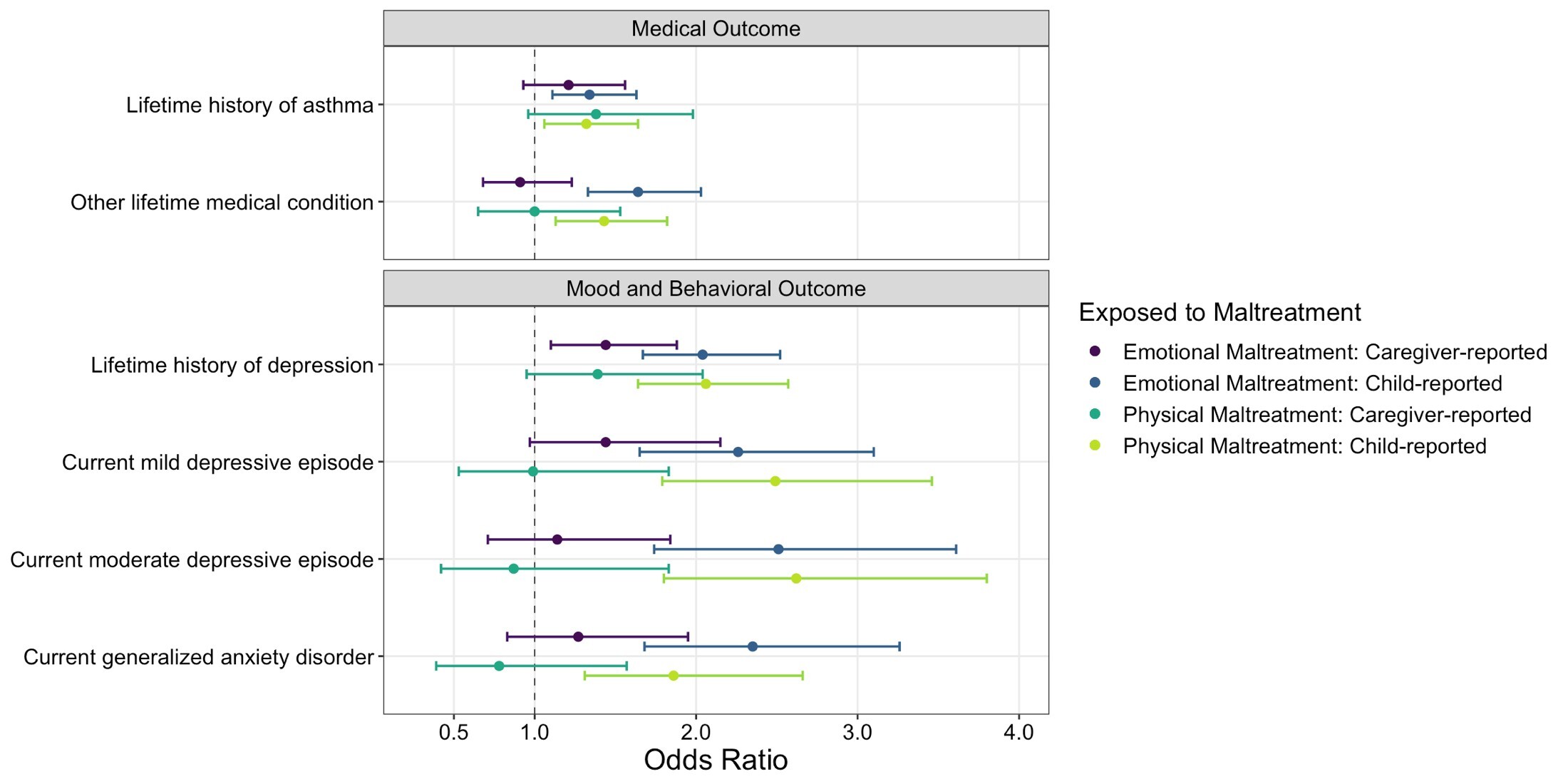 |
| B. Continuous outcomes. |
| 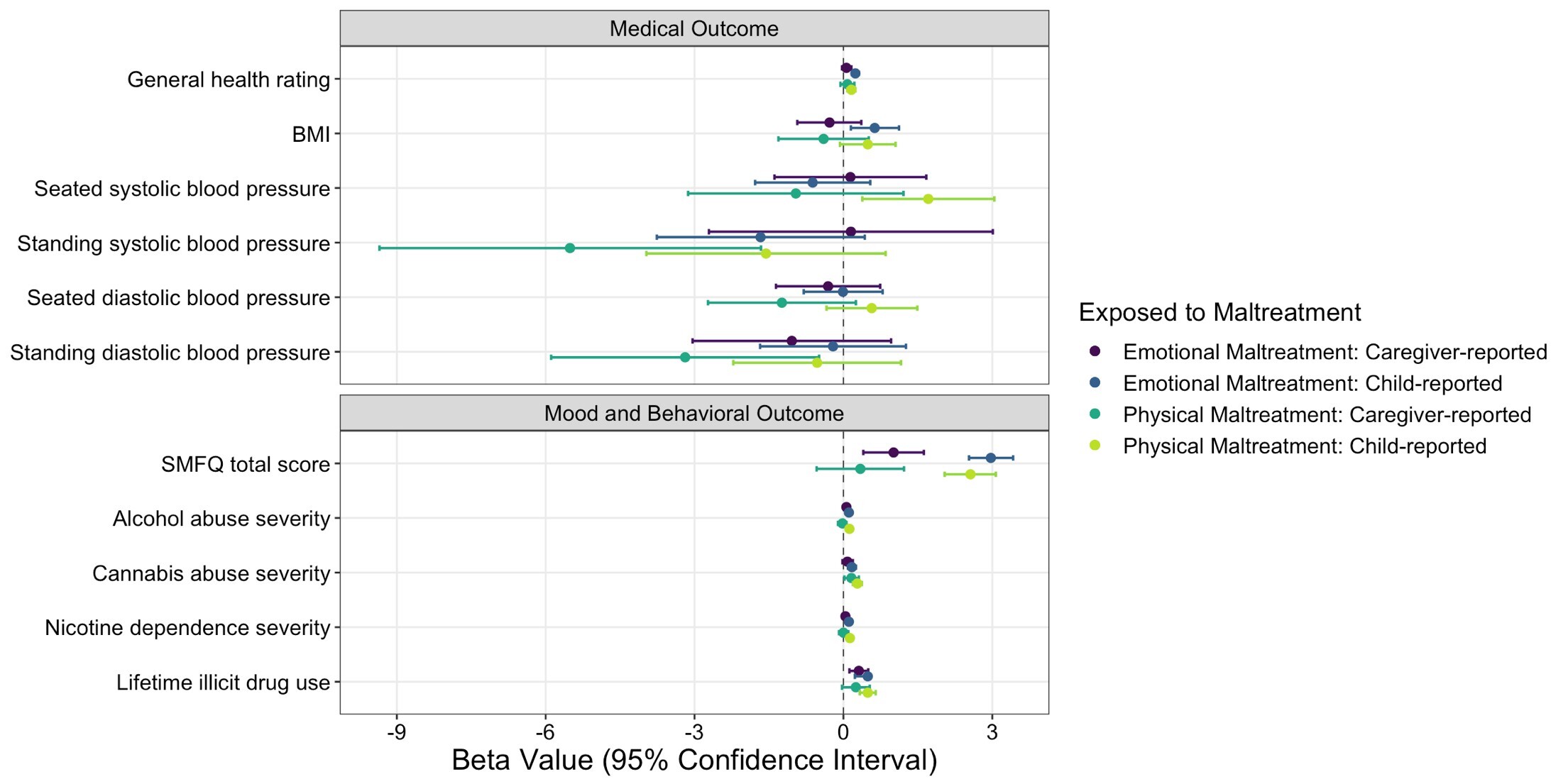 |

**References**

1. Boyd A, Golding J, Macleod J, et al. Cohort Profile: The ‘Children of the 90s’—the index offspring of the Avon Longitudinal Study of Parents and Children. *International Journal of Epidemiology*. 2013;42(1):111-127. doi:10.1093/ije/dys064

2. Fraser A, Macdonald-Wallis C, Tilling K, et al. Cohort Profile: the Avon Longitudinal Study of Parents and Children: ALSPAC mothers cohort. *Int J Epidemiol*. Feb 2013;42(1):97-110. doi:10.1093/ije/dys066

3. Northstone K, Lewcock M, Groom A, et al. The Avon Longitudinal Study of Parents and Children (ALSPAC): an update on the enrolled sample of index children in 2019. *Wellcome Open Research*. 2019;4:51. doi:10.12688/wellcomeopenres.15132.1

4. Northstone K, Ben Shlomo Y, Teyhan A, et al. The Avon Longitudinal Study of Parents and children ALSPAC G0 Partners: A cohort profile [version 1; peer review: awaiting peer review]. *Wellcome Open Research*. 2023;8(37)doi:10.12688/wellcomeopenres.18782.1

5. Harris PA, Taylor R, Thielke R, Payne J, Gonzalez N, Conde JG. Research electronic data capture (REDCap)--a metadata-driven methodology and workflow process for providing translational research informatics support. *J Biomed Inform*. Apr 2009;42(2):377-81. doi:10.1016/j.jbi.2008.08.010

6. Bernstein DP, Stein JA, Newcomb MD, et al. Development and validation of a brief screening version of the Childhood Trauma Questionnaire. *Child Abuse Negl*. Feb 2003;27(2):169-90. doi:10.1016/s0145-2134(02)00541-0

7. Koss MP, Gidycz CA. Sexual experiences survey: reliability and validity. *J Consult Clin Psychol*. Jun 1985;53(3):422-3. doi:10.1037//0022-006x.53.3.422

8. Everson MD, Smith JB, Hussey JM, et al. Concordance between adolescent reports of childhood abuse and Child Protective Service determinations in an at-risk sample of young adolescents. *Child Maltreat*. Feb 2008;13(1):14-26. doi:10.1177/1077559507307837

9. Negriff S, Schneiderman JU, Trickett PK. Concordance Between Self-Reported Childhood Maltreatment Versus Case Record Reviews for Child Welfare-Affiliated Adolescents. *Child Maltreat*. Feb 2017;22(1):34-44. doi:10.1177/1077559516674596

10. Houtepen LC, Heron J, Suderman MJ, Tilling K, Howe LD. Adverse childhood experiences in the children of the Avon Longitudinal Study of Parents and Children (ALSPAC). *Wellcome Open Res*. 2018;3:106. doi:10.12688/wellcomeopenres.14716.1

11. Cox JL, Holden JM, Sagovsky R. Detection of postnatal depression. Development of the 10-item Edinburgh Postnatal Depression Scale. *The British Journal of Psychiatry*. Jun 1987;150:782-6.

12. Matthey S, Henshaw C, Elliott S, Barnett B. Variability in use of cut-off scores and formats on the Edinburgh Postnatal Depression Scale: implications for clinical and research practice. *Arch Womens Ment Health*. Nov 2006;9(6):309-15. doi:10.1007/s00737-006-0152-x

13. Bowen E, Heron J, Steer C. El Komy M, ed. *Anti-social and other problem behaviors among young children: Findings from the Avon Longitudinal Study of Parents and Children*. Home Office Online; 2008.

14. Baker M, Stabile M, Deri C. What Do Self-Reported, Objective, Measures of Health Measure? *The Journal of Human Resources*. 2004;39(4):1067-1093. doi:10.2307/3559039

15. Onur I, Velamuri M. The gap between self-reported and objective measures of disease status in India. *PLoS One*. 2018;13(8):e0202786. doi:10.1371/journal.pone.0202786

16. Angold A, Costello EJ, Messer SC, Pickles A. Development of a short questionnaire for use in epidemiological studies of depression in children and adolescents. *International Journal of Methods in Psychiatric Research*. 1995 1995;5(4):237-249.

17. Lewis G, Pelosi AJ, Glover E, et al. The development of a computerized assessment for minor psychiatric disorder. *Psychol Med*. Aug 1988;18(3):737-45. doi:10.1017/s0033291700008448

18. American Psychiatric Association. *Diagnostic and statistical manual of mental disorders*. 5th ed. American Psychiatric Publishing; 2013.

19. Legleye S, Karila L, Beck F, Reynaud M. Validation of the CAST, a general population Cannabis Abuse Screening Test. *Journal of Substance Use*. 2007/01/01 2007;12(4):233-242. doi:10.1080/14659890701476532

20. Heatherton TF, Kozlowski LT, Frecker RC, Fagerström KO. The Fagerström Test for Nicotine Dependence: a revision of the Fagerström Tolerance Questionnaire. *Br J Addict*. Sep 1991;86(9):1119-27. doi:10.1111/j.1360-0443.1991.tb01879.x

21. Cornish RP, Macleod J, Boyd A, Tilling K. Factors associated with participation over time in the Avon Longitudinal Study of Parents and Children: a study using linked education and primary care data. *International Journal of Epidemiology*. 2020;50(1):293-302. doi:10.1093/ije/dyaa192

22. Taylor AE, Jones HJ, Sallis H, et al. Exploring the association of genetic factors with participation in the Avon Longitudinal Study of Parents and Children. *Int J Epidemiol*. Aug 1 2018;47(4):1207-1216. doi:10.1093/ije/dyy060
